# Comparison of Alcian Blue, Trypan Blue, and Toluidine Blue for Visualization of the Primo Vascular System Floating in Lymph Ducts

**DOI:** 10.1155/2015/725989

**Published:** 2015-08-25

**Authors:** Da-Un Kim, Jae Won Han, Sharon Jiyoon Jung, Seung Hwan Lee, Richard Cha, Byung-Soo Chang, Kwang-Sup Soh

**Affiliations:** ^1^Nano Primo Research Center, Advanced Institute of Convergence Technology, Seoul National University, Suwon 443-270, Republic of Korea; ^2^College of Physical Education, The University of Suwon, Hwaseong 445-743, Republic of Korea; ^3^Department of Cosmetology, Hanseo University, Seosan 356-706, Republic of Korea

## Abstract

The primo vascular system (PVS), floating in lymph ducts, was too transparent to be observed by using a stereomicroscope. It was only detectable with the aid of staining dyes, for instance, Alcian blue, which was injected into the lymph nodes. Some dyes were absorbed preferentially by the PVS than the lymph wall. It remains a standing problem to know what dyes are absorbed better by the PVS than the lymph walls. Such information would be useful to unravel the biochemical properties of the PVS that are badly in need for obtaining large amount of PVS specimens. In the current work we tried two other familiar dyes which were used in PVS research before. We found that Trypan blue and toluidine blue did not visualize the PVS. Trypan blue was cleared by the natural washing. Toluidine blue did not stain the PVS, but it did leave stained spots in the lymph wall and its surrounding tissues, and it leaked out of the lymph wall to stain surrounding connective tissues. These completely different behaviors of the three dyes were found for the first time in the current work and provide valuable information to elucidate the mechanism through which some special dyes stained the PVS preferentially compared to the lymphatic wall.

## 1. Introduction

Long threadlike structures floating in large-caliber lymph ducts were first observed with the aid of staining with the dye Janus green B in the lymph ducts stemming from the lumbar nodes near the caudal vena cava in the abdominal cavity of a rabbit [1]. Since then, various other dyes, such as fluorescent magnetic nanoparticles, Alcian blue [2, 3], and DiI [4], have been used for the same purpose with similar protocols [5, 6]. Subject animals were rabbits [1–5], rats [6], and mice [7]. The target lymph ducts were thoracic ducts [7], as well as the above-mentioned lumbar duct, and the duct around the epigastric blood vessel in the skin [8, 9]. The presence of a lymph-associated primo vascular system (L-PVS) floating in the flow of lymph was first claimed by Kim in the early 1960s [10, 11] and was only recently confirmed by various experimental groups [1–9, 12].

One of the important questions concerns the mechanism through which the dye preferentially stains the L-PVS rather than the lymph wall. In answering this question, knowing which dyes do not stain the L-PVS in a similar process would be helpful. If L-PVS affine and nonaffine dyes are compared, valuable information on the mechanism of the staining can be obtained. The current work reports on the findings for two dyes, Trypan blue and toluidine blue, that did not stain the L-PVS when the same protocol as that for the Alcian blue was used. In the first case, we injected Alcian blue into the inguinal node of one side and Trypan blue into the inguinal node of the other side. In the second case, toluidine blue and Alcian blue were compared. Trypan blue was chosen for this study because it is effective for staining the primo vascular system (PVS) on the surfaces of internal organs [13], in the brain and the spine [14], and around cancer tissues [15–18]. In the second case, toluidine blue was chosen for this study simply because it is one of the common dyes used in histology and has been used for PVS histology in connection with transmission electron microscopy [19].

Effectively visualizing, observing, and isolating the L-PVS is one of the steps necessary to establish the anatomy and the histology of the L-PVS. The current work is one of various efforts with that purpose. The present situation of PVS research is similar to that of the lymph system about a decade ago. Before the first breakthrough with the identification of the vascular endothelial growth factor VEGF-C as a specific lymphangiogenic growth factor, only a rudimentary understanding of the molecular biology of lymphatics existed due to technical difficulties in identifying lymph vessels within tissues and in isolating pure cultures of lymphatic endothelial cells for detailed characterization [20–22]. A molecular understanding of the PVS, which, in turn, requires a sufficient amount of a specimen for study, is badly needed. The current work is connected indirectly to ways to obtain sufficient specimens of the L-PVS.

The possible medical significance of the PVS was suggested by the observation of abundant immune cells in the PVS with the following population ratios: mast cells (20%), eosinophils (16%), neutrophils (15%), lymphocytes (1%), immature cells (3%), and chromatin cells (0.3%) [12]. These data suggest the roles of the PVS in the immune system and in the well-known lymphatic system. The L-PVS, especially, is interesting because it resides inside lymph ducts, thus indicating a probable interaction between them. As is well known, lymphatics are major paths for metastasis in common cancers, such as breast cancer and colorectal carcinomas, which initially occurs via lymph nodes [23, 24]. Strong evidence exists that the PVS is another path for metastasis in addition to the lymphatic one [15]. Furthermore, a primo node might play a role as a haven for cancer stem cells [18].

## 2. Materials and Methods

### 2.1. Animals

Rats (Sprague-Dawley, male, 9 weeks old, 280~300 g) were obtained from DooYeol Biotech (Seoul, Republic of Korea) and housed in a temperature-controlled environment (23°C). Seventeen rats were injected with Trypan blue and twenty-one rats were injected with toluidine blue in the experiments. All animals were exposed to a 12-hour light-dark cycle and were provided food and water* ad libitum*. The procedures involving the animals and their care were in full compliance with current international laws and policies (*Guide for the Care and Use of Laboratory Animals*, National Academy Press, 1996) and were approved by the Institutional Ethics Committee of the Advanced Institute of Convergence Technology (Approval Number: WJIACUC20130212-1-07). The rats were anesthetized by intramuscular injection of a regimen consisting of 1.5 g/kg of urethane and 20 mg/kg of xylazine.

### 2.2. Staining Dye Preparation

The staining dye 0.2% Alcian blue (A5268, Sigma-Aldrich, St. Louis, MO, USA) solution in boiled phosphate-buffered saline (PBS, pH 7.4) was prepared and was filtered by using a 0.22-*μ*m membrane filter (Merck Millipore, Darmstadt, Germany) with a syringe (BD, Franklin Lakes, NJ, USA). The staining dye Trypan blue solution (25900048R, CORNING Cellgro, Manassas, VA 20109, USA) 0.4% (w/v) in PBS was used. The staining dye with a fiducial volume of 50% toluidine blue working solution was made from the stock solution and 1% sodium chloride (NaCl: 0.05 gm and distilled water: 5 mL). The toluidine blue stock solution was made by melting 0.1 gm of toluidine blue powder (Toluidine blue O, 198161-5G, Sigma-Aldrich, St. Louis, MO, USA) and 10 mL of 70% alcohol.

### 2.3. *In-Vivo *Surgical Operation and Visualization of the Primo Vascular System

A 2 cm skin midline incision up and down from the navel along the ventral surface of the abdominal cavity was made, and the skin was retracted towards the rat's spinal column. The inguinal nodes (INs) are bilateral and are situated close to the bifurcation of the superficial epigastric veins. After the left-hand side IN had been exposed, the prepared 0.2% Alcian blue dye was injected into the node at a slow rate. Then, Trypan blue was injected into the right-hand side IN. The order of injections was varied and the left and the right sides were alternated from one experiment to another. The other experiments with Alcian blue and toluidine blue were done using similar procedures.

The main lymph duct to observe was located along the superior epigastric vein and connected the IN to the axillary node (AN); most of the time, it had several branches. Natural circulation of the lymph fluid had to be promoted in order to ensure that the dyeing agent had been thoroughly washed out. Therefore, each rat was covered with paper tissue and kept in the bedding to maintain its body temperature.

Two hours after dye injection, the IN and the lymph duct were exposed to observe the L-PVS. All procedures were performed under a stereomicroscope (SZX12, Olympus, Japan). After the stained L-PVS had been observed, the rats were sacrificed by using an intracardiac injection of urethane (1 mL). The skin, including the lymph ducts, was fixed with a 10% neutral buffered formalin (NBF) solution at 4°C for 24 hours. The lymph ducts, including the L-PVS, were collected from the fixed skin.

For the staining of nuclei with 4′, 6-diamidino-2-phenylindole (DAPI), the specimen was stained with 300-nM DAPI (D1306, Invitrogen, MO, USA) solution for 20 minutes. After the specimen had been washed, it was covered with a mounting solution. The stained specimen was investigated under a phase contrast microscope (Olympus, U-LH100HG, Japan), a microscope with a black and white charge-coupled device (CCD; Nikon, ECLIPSE Ti, Japan), a contrast-enhanced microscope (DM2500, Leika, Germany), and a confocal laser scanning microscope (CLSM; C1 plus, Nikon, Japan).

## 3. Results

**Figure 1:**
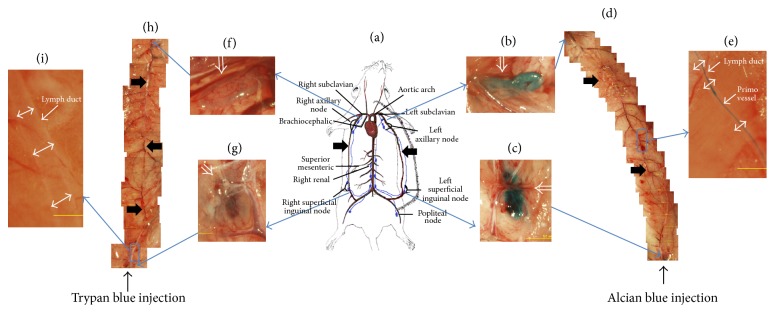
Stereomicroscopic images of lymph ducts in which Alcian blue and Trypan blue had been injected. (a) Illustration of the left and the right lymph ducts along the epigastric blood vessels (thick arrows) in skin. (b) Left axillary node (open arrow) which became blue due to the Alcian blue that flowed in the lymph duct. (c) Left inguinal node (open arrow) into which Alcian blue had been injected. (d) Mosaic of images of the left lymph duct along the epigastric blood vessel (thick arrows). (e) Magnified image of the rectangular area in (d). The blue threadlike structure was the primo vessel (arrow), and it was floating in the lymph duct (double arrows). (f) Right axillary node (open arrow) weakly stained by Trypan blue that flowed from the inguinal node through the lymph duct. (g) Right inguinal node (open arrow) into which Trypan blue had been injected. (h) Mosaic images of the right lymph duct from the inguinal node to the axillary node in which Trypan blue flowed. The epigastric blood vessel is indicated with thick arrows. (i) Magnified image of the rectangular area in (h). The lymph duct (double arrows) was washed clean, and the PVS was not stained. More details are presented in Figure 3.

The position of the relevant lymph duct in the skin from the inguinal node to the axillary node along the prominent epigastric blood vessel is illustrated in Figure 1(a). As in a previous work with Alcian blue [8], the PVS was observed in this skin lymph duct. In Figure 1(b), the left axillary node was blue due to the Alcian blue which had been injected into the left inguinal node (Figure 1(c)) and had arrived via the lymph duct. A mosaic of the images capturing the whole lymph duct (Figure 1(d)) shows the primo vessel, where one part (Figure 1(e)) was magnified to demonstrate that the primo vessel was stained well by Alcian blue inside the lymph vessel. In comparison, the right axillary node (Figure 1(f)) became weakly blue due to the Trypan blue that had been injected into the right inguinal node (Figure 1(g)) and had flowed via the lymph duct (Figure 1(h)). The magnified mosaic image of the lymph duct (Figure 1(i)) showed that the primo vessel was not stained at all. This showed that Trypan blue in this injection method was not effective for L-PVS visualization.

**Figure 2:**
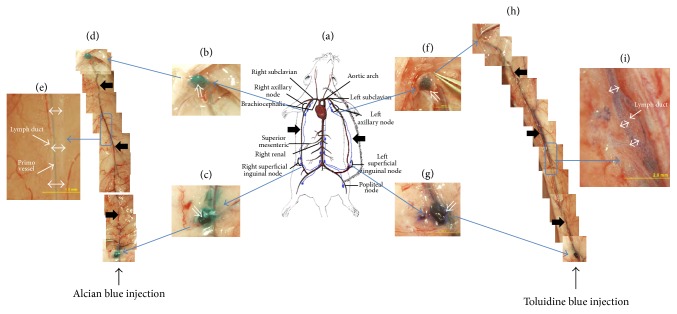
Stereomicroscopic images of lymph ducts in which Alcian blue and toluidine blue had been injected. (a) Illustration of the left and the right lymph ducts along the epigastric blood vessels in skin (thick arrows). (b) Right axillary node which became blue due to the Alcian blue that flowed through the lymph duct. (c) Right inguinal node into which Alcian blue had been injected. (d) Mosaic of images of the right lymph duct along the epigastric blood vessel (thick arrows). (e) Magnified image of the rectangular area in (d). The blue threadlike structure was the primo vessel, and it was floating in the lymph duct (double arrows). (f) Left axillary node stained with toluidine blue that flowed from the inguinal node through the lymph duct. (g) Left inguinal node into which toluidine blue had been injected. (h) Combined images of the right lymph duct from the inguinal node to the axillary node in which toluidine blue flowed. (i) Magnified image of the rectangular area in (h). The lymph duct (double arrows) and its surrounding tissue were stained blue, but the PVS was not visible. More details are presented in Figure 4.

Compared with Alcian blue and Trypan blue, the dye toluidine blue showed a completely different behavior, as shown in Figure 2. Figure 2(a) is the same illustration of the rat lymph in skin, and the panels from Figures 2(b) to 2(e) are for the right axillary node and the right inguinal node and present a mosaic of images of the lymph duct, along with one magnified image showing the primo vessel in the cleared lymph duct, respectively. Alcian blue showed no differences in its effectiveness in comparison with Trypan blue and toluidine blue. The results with toluidine blue are depicted in Figures 2(f) to 2(i). The left axillary and inguinal nodes were deeply colored due to toluidine blue (Figures 2(f) and 2(g)). The magnified images in Figures 2(h) and 2(i) show that the L-PVS was not stained, but the lymph duct was stained at randomly distributed spots in its wall; furthermore, the dye leaked out to the surrounding tissue.

**Figure 3:**
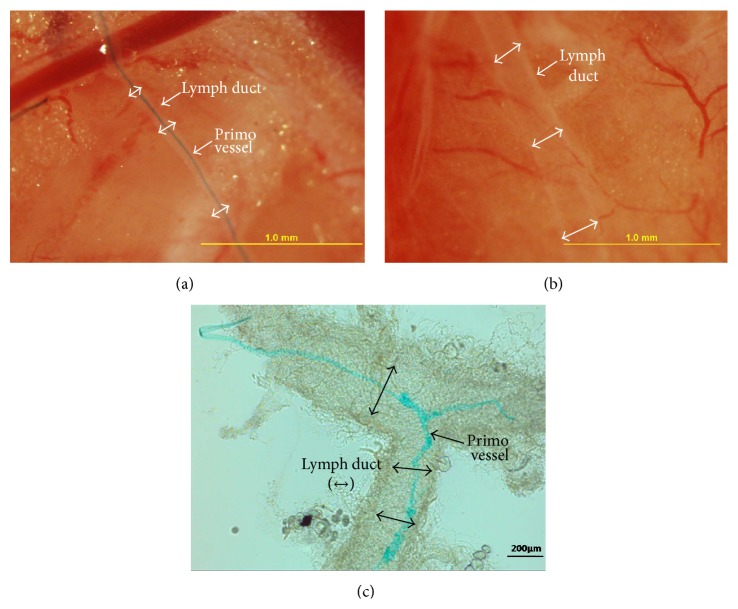
Comparison of stereomicroscopic images of the lymph ducts into which Alcian blue and Trypan blue had been injected. (a) A primo vessel emerged in a lymph duct (double arrows) after a two-hour washing of the injected Alcian blue. This image corresponds to Figure 1(e). (b) The lymph duct after a two-hour washing of the injected Trypan blue became clear again without any hint of the presence of the primo vessel. This showed that the L-PVS was selectively stained by the Alcian blue, not by the Trypan blue. (c) Contrast-enhanced microscope image of a branched lymph duct (double arrows) that was extracted and put on a slide. Connective tissues wrapped the lymph duct. The stained L-PVS also branched.

Figure 3(a) shows the magnified images of the primo vessel inside the lymph duct.  Figure 3(b) shows the transparent lymph duct after washing of the preinjected Trypan blue. The L-PVS was not stained at all, so it was not observable.  Figure 3(c), an enhanced contrast microscope image of the specimen on a slide glass, shows an extracted lymph duct with the Alcian-blue-stained primo vessel in it. A small part of the primo vessel was drawn out on the left upper part of the lymph duct. This demonstrates the existence of an independent threadlike object inside the lymph duct. Also, the primo vessel is seen to branch where the lymph duct branched.

**Figure 4:**
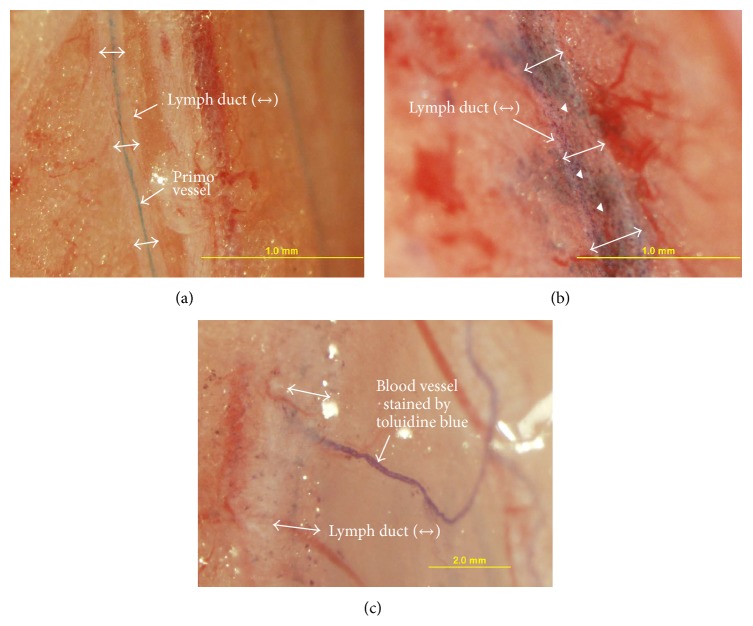
Comparison of lymph ducts into which Alcian blue and toluidine blue had been injected. (a) Two branches of lymph ducts (one of them indicated with double arrows) and their L-PVS stained with Alcian blue. (b) Toluidine blue left stained spots in the lymph wall (arrow heads). It also leaked from the wall and stained the surrounding tissues. However, the L-PVS was not stained. (c) A thin blood vessel was deeply stained by using toluidine blue. How it flowed into the blood vessel is not known.

Figure 4(a) shows another Alcian-blue-stained primo vessel in comparison with the toluidine blue injection case (Figure 4(b)) in which no primo vessel was observed, but in which the lymph duct and the surrounding tissue were weakly stained. In addition, strongly stained spots were sporadically distributed. The dye leaked from the lymph duct. Furthermore, some small blood vessels were strongly stained, and apparently the dye flowed in the blood vessel (Figure 4(c)). This leaking from the lymph duct and flowing in blood vessels of toluidine blue were observed for the first time in this work.

**Figure 5:**
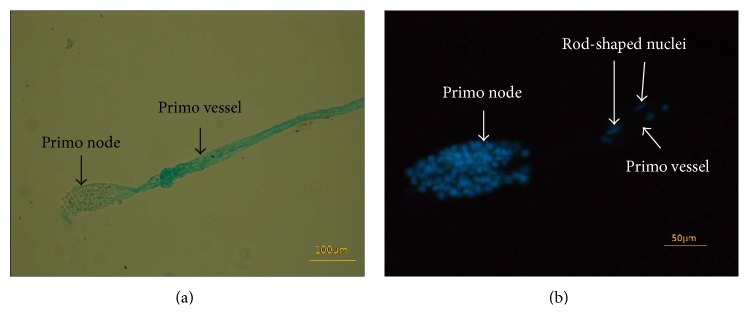
Images of a primo node extracted from the lymph duct. (a) A primo node stained by Alcian blue was extracted from a lymph vessel. One of the primo vessels connected to the node was cut off during the extraction process, and only one side was kept. The primo vessel was a bundle of two or more subvessels. (b) DAPI images of the same primo node as in (a), which contained many nuclei. Two rod-shaped nuclei (arrows) can be seen in the primo vessel.

**Figure 6:**
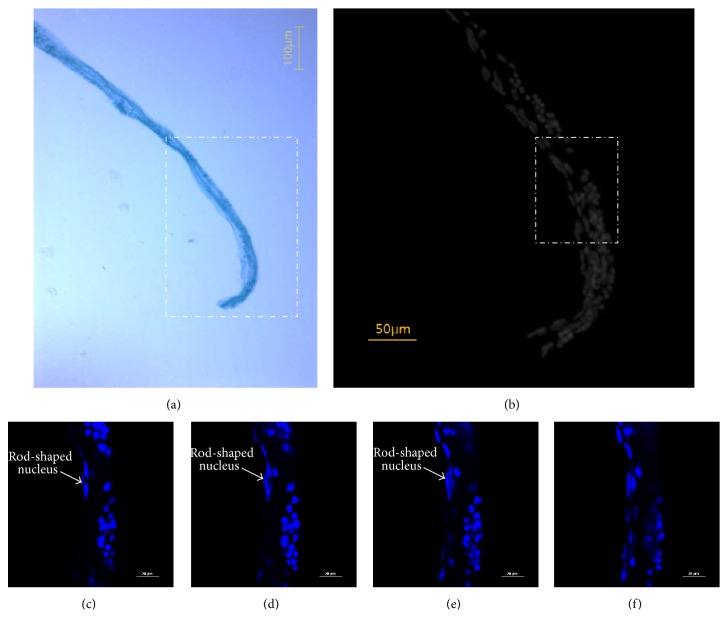
Rod-shaped nuclei in the primo vessel. (a) Phase contrast microscope image of a primo vessel that was extracted from a lymph duct. (b) Fluorescence microscopic image of the boxed area in (a). The boxed area was magnified, and the part with rod-shaped nuclei was the primo vessel. The round-shaped nuclei were aggregated lymphocytes. (c–f) Confocal laser scanning microscope images of the circled area in (b). These panels were consecutive optical sections from top to bottom separated by 2 *μ*m in each step. The same rod-shaped nucleus appeared (panel (c)), looked long (panels (d) and (e)), and then nearly disappeared (panel (f)), which implies that this particular endothelial nucleus was inside the primo vessel and, therefore, had not aggregated from outside.

The primo node is an oval-shaped tissue packed with various cells [12].  Figure 5 shows a primo node attached to one primo vessel, during preparation. Indeed, the primo node was packed with various cell nuclei, as seen in Figure 5(b). The minimal criterion to confirm the presence of the L-PVS is the observation of a distribution of the rod-shaped nuclei by DAPI staining. Figure 5(b) shows the presence of longitudinally distributed rod-shaped nuclei; more are shown in Figure 6.

Because the distribution of nuclei and their shapes are critical, we present one more specimen with DAPI staining. A phase contrast microscopic image of the extracted primo vessel is shown in Figure 6(a). The DAPI image was taken with a sensitive black and white CCD (Figure 6(b)) and distinguished the part with the rod-shaped nuclei from the part with the round-shaped nuclei of aggregated lymphocytes. The rod-shaped nuclei were located inside the primo vessel, which was confirmed by a series of confocal microscope images of optical sections (Figures 6(c) to 6(f)). For verification, we chose a particular nucleus (arrow in Figures 6(d) and 6(e)).

## 4. Discussion

To the present time, several dyes have been used for the visualization of the L-PVS in lymph ducts: Janus green B [1], fluorescent magnetic nanoparticles, Alcian blue [2, 3, 5, 6, 8], and DiI [4]. Among them, Alcian blue has been most extensively used. Even though all these dyes are effective and have different merits, the mechanism through which they stain preferentially the L-PVS has not yet been uncovered. Before we attempted to determine the exact mechanism, we examined the efficacies of two common materials for the visualization of the L-PVS, the results of which should provide more information useful in investigating the mechanism.

Alcian blue, Trypan blue, and toluidine blue showed different behaviors in this experiment. Alcian blue is used for the staining of acidic polysaccharides in histology [25]. It was initially chosen with the expectation that it would stain the hyaluronic acid that is known to be abundant in the primo fluid [11]. Until now, whether the successful application of Alcian blue in many previous experiments [2, 3, 5, 6, 8] was due to its affinity to hyaluronic acid is not yet clear. In the current work, Alcian blue was used as a reference material for the Trypan blue and toluidine blue.

The dye Trypan blue is used in histology for staining connective tissues, such as collagen, muscle, and cornified epithelium [25]. Another common use is to discriminate dead cells from live ones. Trypan blue is also useful for vivi-staining of vitereoretinal membranes in ophthalmic surgery [26]. In PVS-visualization work, Trypan blue was first used as a visualizing agent* in vivo *and* in situ*, not as a staining dye of tissue specimens for histological purposes. By using it, we were able to make the following significant contributions: (1) a web-like network of PVS was observed on the omentum and the visceral peritoneum [13]; (2) the PVS exists around cancer tissues [15–18]; and (3) the PVS exists in the brain and the spine [14]. In the current work, we compared Alcian blue and Trypan blue for their abilities to visualize the L-PVS. Surprisingly, the Trypan blue was not absorbed either by the lymph wall or the L-PVS. We, therefore, conjecture that Trypan blue might have been absorbed by the dead cells of the PVS, which had been exposed to air during the experimental procedure. However, further study is needed before a definite conclusion can be drawn about the mechanism of Trypan-blue absorption by the PVS in previous experiments.

The behavior of the toluidine blue was beyond our expectation in the sense that it leaked out through the lymphatic wall and left randomly-distributed dots in the lymph wall and its surrounding tissues. In addition, the dye flowed in some venules around the lymph duct and strongly stained the vascular wall, but it was not absorbed by the L-PVS. These results were newly found in this experiment. Toluidine blue has often been used in the staining of PVS tissues for light microscopic imaging in transmission electron microscopic studies of PVS tissues [19].

The possible medical significance of the PVS is suggested by the observation of abundant immune cells in the PVS [12]. The data suggest the roles of the PVS in immune systems and as an extrahematopoietic source of immune cells and bone marrow. Other suggested functions of the PVS are a path for metastasis [15] and a haven for cancer stem cells [18], which is worth deeper study.

The present work was limited in that it only compared the efficacies of the three dyes in visualizing the L-PVS and did not uncover the biochemical mechanism for their different behaviors. The understanding of this mechanism is left for a future study. For this purpose it is valuable to know the dyes that were used to stain lymph vessels. For example, Evans blue, and guanylate cyclase that was used to stain dermal lymphatic capillary [27].

In conclusion, Trypan blue and toluidine blue were quite different from Alcian blue in their abilities to visualize the L-PVS. Trypan blue was cleared completely by a two-hour natural washing and did not stain either the lymph wall or the L-PVS. To the contrary, toluidine blue left scattered strongly stained dots in the lymph wall and surrounding tissues after leaking out from the lymph duct. It did not, however, stain the L-PVS at all. Sometimes, it entered some small veins and stained the vessel strongly. These were first observed in this experiment, but this phenomenon, in terms of biochemical processes, is still not understood.
